# Adopting World Health Organization Multimodal Infection Prevention and Control Strategies to Respond to COVID-19, Kenya

**DOI:** 10.3201/eid2813.212617

**Published:** 2022-12

**Authors:** Daniel Kimani, Linus Ndegwa, Mercy Njeru, Eveline Wesangula, Frankline Mboya, Catherine Macharia, Julius Oliech, Herman Weyenga, George Owiso, Kamau Irungu, Ulzii-Orshikh Luvsansharav, Amy Herman-Roloff

**Affiliations:** US Centers for Disease Control and Prevention, Nairobi, Kenya (D. Kimani, L. Ndegwa, M. Njeru, F. Mboya, J. Oliech, H. Weyenga, A. Herman-Roloff);; Ministry of Health, Nairobi (E. Wesangula, K. Irungu); US Department of Health and Human Services, Nairobi (C. Macharia);; International Training and Education Center for Health, Nairobi (G. Owiso);; Centers for Disease Control and Prevention, Atlanta, Georgia, USA (U.-O. Luvsansharav)

**Keywords:** COVID-19, pandemic, SARS-CoV-2, infection control, World Health Organization, coronavirus disease, severe acute respiratory syndrome coronavirus 2, viruses, respiratory infections, zoonoses, Kenya

## Abstract

The World Health Organization advocates a multimodal approach to improving infection prevention and control (IPC) measures, which Kenya adopted in response to the COVID-19 pandemic. The Kenya Ministry of Health formed a national IPC committee for policy and technical leadership, coordination, communication, and training. During March–November 2020, a total of 69,892 of 121,500 (57.5%) healthcare workers were trained on IPC. Facility readiness assessments were conducted in 777 health facilities using a standard tool assessing 16 domains. A mean score was calculated for each domain across all facilities. Only 3 domains met the minimum threshold of 80%. The Ministry of Health maintained a national list of all laboratory-confirmed SARS-CoV-2 infections. By December 2020, a total of 3,039 healthcare workers were confirmed to be SARS-CoV-2–positive, an infection rate (56/100,000 workers) 12 times higher than in the general population. Facility assessments and healthcare workers' infection data provided information to guide IPC improvements.

COVID-19, caused by SARS-CoV-2, emerged in China in December 2019 and quickly spread globally (*1*,*2*). Within a year, >79.2 million persons were infected and >1.7 million persons had died (*3*). In Kenya, the first case was confirmed in March 2020; by December 2020, a total of 96,458 cases and 1,670 deaths had occurred (*4*).

In response to the pandemic, the World Health Organization (WHO) released infection prevention and control (IPC) guidelines in March 2020 for preventing SARS-CoV-2 transmission during healthcare (*5*). WHO recommended that each health facility have a dedicated trained team or IPC focal person to implement basic IPC measures for protection of patients and healthcare workers (*6*).

In 2017, WHO recommended an evidence-based multimodal IPC strategy to address leadership, resources, and training gaps for more effective IPC programs (*7*,*8*). This strategy uses a combination of approaches to achieve the desired behavior change and quality improvement (*6*). The strategy has 5 elements: 1) system change to enable IPC practices; 2) training and education; 3) monitoring and feedback; 4) reminders and communications; and 5) culture of safety.

In response to COVID-19, the Kenya Ministry of Health (MOH) put in place a national COVID-19 task force with several technical committees, one of which was IPC. The MOH tasked the IPC committee with developing strategies to prevent and control the spread of COVID-19 in health facilities and among the public. To respond quickly, the committee decided to build on an existing IPC program within the MOH’s Division of Patient and Healthcare Worker Safety. This division oversaw the development and dissemination of IPC-related guidelines, policies, and strategic plans; implementation of IPC training and surveillance activities; and formation of IPC committees (*9*). Kenya has 47 subnational governments (counties) with a structure mirroring the national level. The national IPC program supported the county programs for activity implementation. We describe how Kenya revised national and county IPC programs to adopt WHO’s multimodal strategies to respond to the COVID-19 pandemic and the outcomes of these efforts in the first 9 months of the pandemic (March–December 2020).

## Methods

### Multimodal Interventions

#### 1. Enabling Environment

The MOH established the IPC committee in March 2020 to provide leadership in IPC implementation across all levels of the healthcare system. The committee met weekly, coordinated work with other COVID-19 committees, and reported to the National Task Force. It advocated that the government and private sector commit resources to create an environment conducive to IPC interventions, including infrastructure improvements, equipment, supplies and staffing.

#### 2. Education and Training

The IPC committee developed a COVID-19 training curriculum for healthcare workers from existing IPC training materials and led a national training-of-trainers (ToT) during March–April 2020. The national trainers trained county trainers who then cascaded the information to health facilities. The training consisted of a comprehensive 3-day practical workshop and an abbreviated 1-day training. Health facilities in areas with high infection risk were prioritized for the 3-day trainings. Training topics included introduction to IPC; standard and additional precautions; donning and doffing of personal protective equipment (PPE); waste management; overview of COVID-19, screening, and management; specimen collection, packaging, and transportation; and surveillance of COVID-19. To avoid group gatherings, the committee implemented biweekly IPC webinars on topics identified as facility gaps. The webinars incorporated subject matter experts, panel discussions, and county presentations to share experiences.

#### 3. Guidelines, Reminders, and Communication

Localized Kenya COVID-19 guidelines, protocols, and information, education, and communication (IEC) materials were developed beginning in March 2020 based on WHO and US Centers for Disease Control and Prevention (CDC) guidelines with an emphasis on standard and respiratory precautions. The committee developed minimum requirements for IPC in COVID-19 quarantine and isolation centers and public health advisories to minimize community transmission.

#### 4. Surveillance, Monitoring, and Feedback

The CDC Facility Readiness Assessment for COVID-19: IPC Considerations in Non-US Healthcare Settings checklist was adopted and modified to fit the Kenya context (*10*). The modified tool had 16 domains, each with a set of questions, possible responses (Yes/No/Not applicable), the assessor’s guide, and a comments section. The questions were scored through healthcare worker interviews or observations at the facility. The domains were coordination, communication/reporting, written IPC/COVID-19 guidelines, hand hygiene supplies/facilities, general IPC supplies, critical IPC supplies, IPC training, screening and triage, COVID-19 patients’ care, preparing for a surge, monitoring healthcare workers, environmental cleaning/disinfection, linen management, handling of COVID-19 cadavers, appropriate mask use, and appropriate glove use. Each domain had a maximum possible score of 100% (Table 1). The team calculated a mean score for each domain across all facilities and set a minimum threshold of 80%. County IPC coordinators were oriented to the tool by the national team and conducted assessments in 777 facilities across the country during July–September 2020. Based on facility-level findings, a work plan was made to address gaps. The work plans were specific: IPC gap identified, activities to address the gap, responsible person, and timeline to close the gap. A national public health emergency operations center (PHEOC) was activated to respond to COVID-19. Data for infected persons from the 43 government-approved SARS-CoV-2 testing laboratories across the country were sent to the PHEOC, which maintained a line list with basic demographic information.

**Table 1:** Infection prevention and control structures activated to respond to COVID-19 at various health system levels, Kenya, 2020*

Level	Structure	Membership and meeting frequency	Function
National	National COVID-19 Response coordination task force	Director general of health, department heads at MOH, WHO, CDC, and other key development partners. Met weekly during March–December 2020	Enhance coordination and leadership for COVID-19 prevention and control
	National COVID-19 IPC committee	Head of patient and healthcare worker unit and IPC team, WHO, CDC, Key IPC partners. Met weekly March–June 2020 then biweekly until December 2020	National coordination and guidance of the IPC interventions, policy and technical leadership on IPC issues
	National COVID-19 training and capacity building committee	MOH and key training partners. Met weekly March–June 2020 then biweekly until December 2020	National coordination of all COVID-19–related trainings and other education initiatives
	Resource mobilization committee	MOH and private sector players supporting PPE and IPC supplies. Met when needed	Mobilizing resources necessary for IPC measures (PPE, IPC supplies)
County	County COVID-19 Response team	County minister of health, county health director, departmental heads. Met weekly March–December 2020	Overall coordination of COVID-19 response at the county level
	County COVID-19 IPC committee	County IPC coordinator, departmental heads. Met monthly or as needed	Coordination of IPC activities in the county
Facility	Facility-level IPC committee	Multidisciplinary team. Met monthly or as needed	Implementation of COVID-19 IPC measures at facility level

*CDC, Centers for Disease Control and Prevention; IPC, infection prevention and control; MOH, Ministry of Health; PPE, personal protective equipment; WHO, World Health Organization.

#### 5. Culture Change

To ensure the culture of safety was rapidly institutionalized, members of the IPC committee sought goodwill from government leaders. Committee members were asked to be agents of change by observing and demonstrating good IPC practices. Messages about COVID-19 were shared through electronic and print media. Healthcare facility administrators were asked to support implementation of IPC measures at the facility level and enhance a culture of safety.

## Results

### Outcomes of Implementing Multimodal Approaches

#### 1. Enabling Environment

The national committee coordinated with county-level IPC committees to support the COVID-19 IPC response. Where no IPC committee existed, a new one was formed (Table 1). The private sector procured and fast-tracked local production of IPC supplies including PPE, hand hygiene supplies, and disinfectants. Through the Equity Group Foundation, 109 local manufacturers were trained to make PPE (*11*). The Kenya Medical Supplies Agency fast-tracked procurement of PPEs and other IPC supplies for distribution to facilities nationally. Separately, counties renovated and modified facility infrastructure to improve ventilation and create additional hand hygiene stations, triage stations, and patient waiting bays to avoid overcrowding and protect healthcare workers. In some facilities, tents were purchased to use as patient waiting bays and temporary holding and isolation areas to ensure adequate distance. The IPC committees at the facility, county, and national levels provided weekly updates on infection rates and emerging gaps to leadership, who in turn committed resources to address them.

#### 2. Education and Training

During March–November 2020, a total of 69,892 (57.5%) of the estimated 121,500 healthcare workers in Kenya at the time were trained on IPC. Of these, 25,999 (37.2%) received the 3-day training, and 43,893 (62.8%) received the 1-day training. COVID-19 biosafety training was provided to 100 laboratory staff from 10 national molecular diagnostic laboratories and 2,058 staff members from county laboratories in preparation for SARS-CoV-2 testing. The teams conducted 10 IPC webinars, reaching an average of 200 participants per session.

#### 3. **Guidelines, Reminders, and Communication**

During March–June 2020, the IPC committee developed or provided input in developing these COVID-19—related guidelines targeting healthcare workers: IPC considerations for healthcare settings, setting up quarantine and isolation centers, health and safety in the workplace, waste management, home-based care, safe handling of human remains, case management, and rational use of PPE. Posters, banners, and brochures with simplified information were developed in English and translated to local languages targeting the public. These materials consisted of information on understanding COVID-19, handwashing, cough etiquette, and home-based care. Public health advisories on proper use of masks and gloves were developed. Materials targeting healthcare workers were hosted on the MOH website and shared through training sessions (*12*). According to health facility assessments, only 52.6% of the facilities had all the documents by September 2020. Materials targeting the public were disseminated through print and electronic media.

#### 4. Surveillance, Monitoring, and Feedback

**Figure 1:**
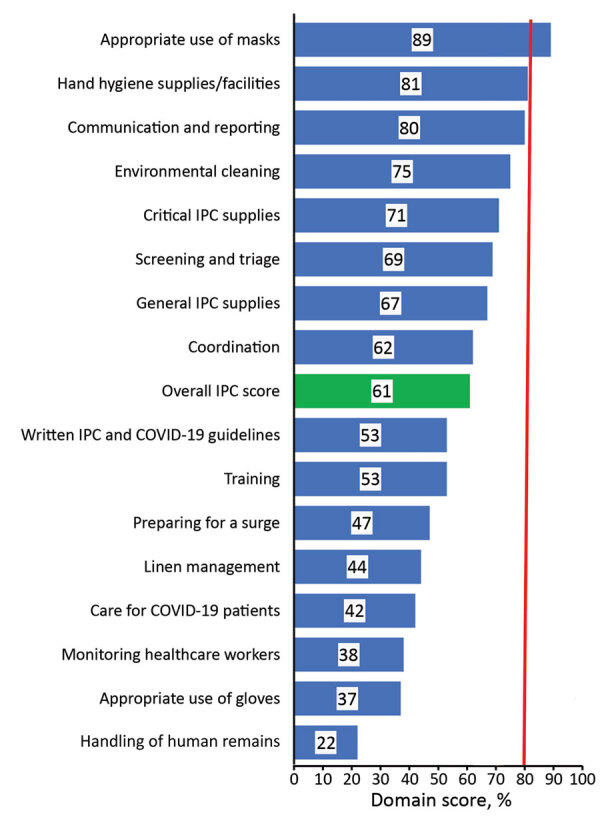
Assessment scores across various domains for IPC readiness assessment among 777 health facilities, Kenya, 2020. Red line indicates optimal score of 80%. IPC, infection prevention and control.

**Figure 2:**
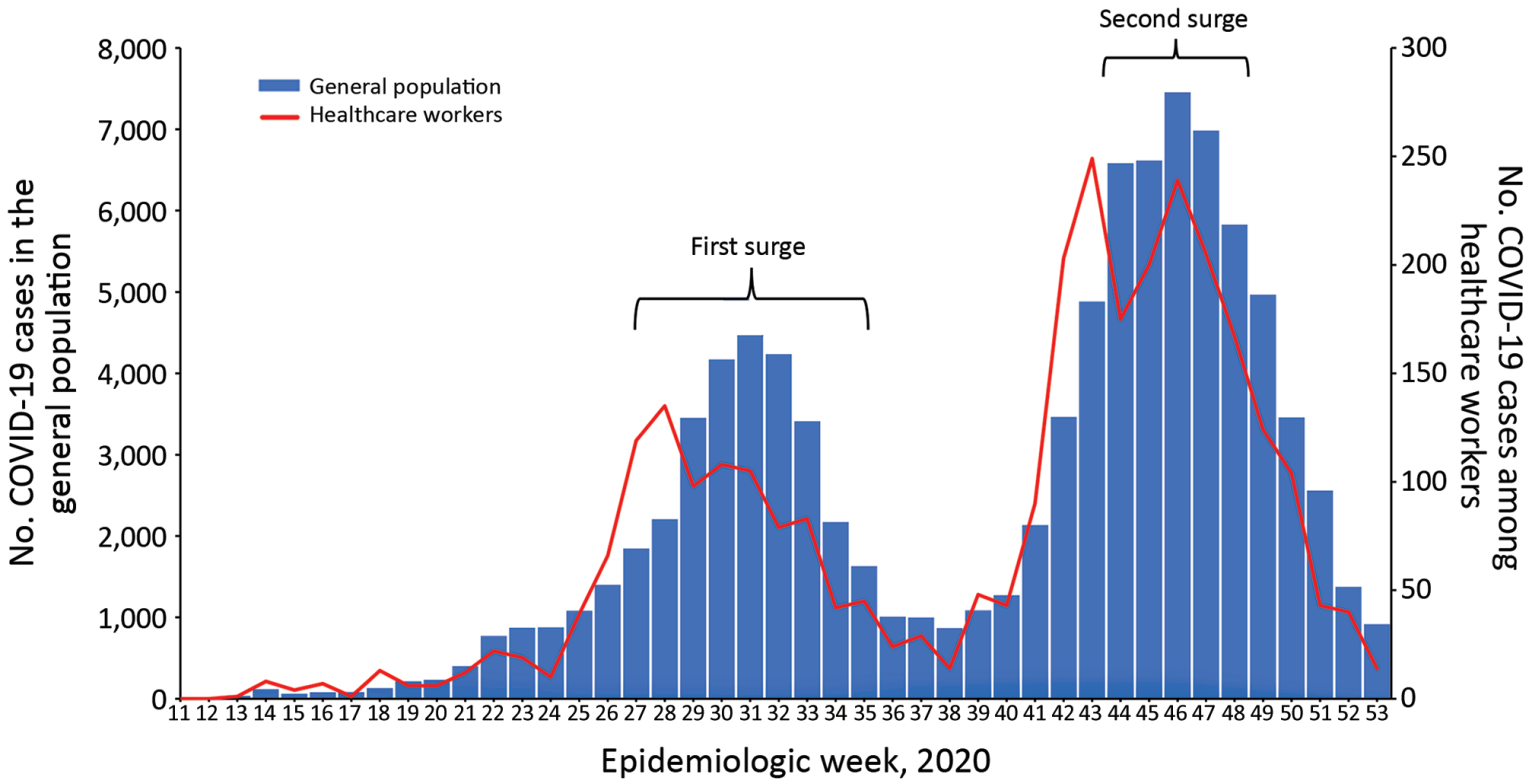
Epidemic curve for COVID-19 in general population and healthcare workers, Kenya, 2020.

According to the health facility assessment, only 3 domains met the minimum threshold mean score of 80% across all facilities. The 3 domains included communication and reporting (80%), availability of hand hygiene supplies and facilities (81%), and appropriate mask use (89%) (Figure 1). The mean score across all domains was 61%; the lowest score was for handling of human remains (22%). The assessments yielded specific recommendations for remediation within each domain (Table 2). By late December 2020, PHEOC data indicated that 96,421 persons, including 3,039 healthcare workers, had laboratory-confirmed COVID-19 (Figure 2). Infections among healthcare workers accounted for 3.2% of all SARS-CoV-2 infections in Kenya. Compared with the general population (4.8/100,000 persons), the infection rate in healthcare workers (56/100,000 workers) was ≈12 times higher. Infections in healthcare workers mirrored the peaks in the general population during the June–August and October–December 2020 surge periods (Figure 2).

**Table 2:** Assessment scores and remediation activities recommended for each COVID-19–related domain assessed in 777 health facilities, Kenya, 2020*

Domain	Score, %	Recommended remedial actions
Appropriate use of face masks	89	Conduct PPE training, provide IEC materials, provide a variety of masks, conduct IPC audits
Hand hygiene	81	Conduct HH training, provide IEC materials, provide HH supplies (soap and alcohol-based hand rub) and renovate/install HH facilities
Communication and reporting	80	Provide IPC/COVID-19 guidelines, develop patient referral algorithms and referral contacts
Cleaning and disinfection	75	Provide disinfectants and other supplies, develop SOPs, and conduct routine audits
Critical supplies	71	Estimate supply needs, train on inventory management, appoint a supply-management lead
Screening and triage	69	Create clear signage, mark patient sitting areas, and provide PPE, screening tools, and data collection tools to the triage nurse
Supplies	67	Estimate supply needs, train on inventory management, appoint a supply-management lead
Coordination	62	Activate IPC committee or appoint IPC focal person, establish a COVID-19 response team
IPC and COVID-19 guidelines	53	Provide updated IPC/COVID-19 guideline and orient healthcare workers on the same
Training	53	Provide in-person and virtual training, webinars, and facility education sessions
Preparing for a surge	47	Define facility capacity, create temporary isolation centers (e.g. tents) and link with home-based care
Management of linen	44	Provide SOPs on linen management, provide supplies, separate isolation linen from others
Care COVID-19 patients	42	Improve patient flow, create donning/doffing areas, develop SOPs on case management/IPC and airborne precautions for aerosol-generating activities
Monitoring healthcare workers	38	Provide healthcare worker risk assessment tools, screening, and monitoring of exposed workers
Appropriate use of gloves	37	Improve training, IEC materials, availability of gloves and HH supplies
Handling of human remains	22	SOPs for body management, training of morticians and those handling bodies

*HH, hand hygiene; IEC, information, education and communication; IPC, infection prevention and control; PPE, personal protective equipment; SOP, standard operating procedure.

#### 5. Culture Change

To ensure consistency in COVID-19 IPC practices, senior leadership in government complied with COVID-19 protocols. Top MOH officials provided daily COVID-19 updates on number of infections and fatalities and continually emphasized key prevention measures. Across electronic and print media, healthcare and political leaders were seen wearing masks, keeping physical distance, and practicing hand hygiene. Most meetings were held virtually, and training events were conducted in open-air environments for good ventilation. In health facilities, patients were required to wear a mask to receive service.

## Discussion

Kenya’s adoption of WHO multimodal strategies in response to the COVID-19 pandemic required a pragmatic approach and appropriate leadership in coordinating multiple stakeholders. Kenya enhanced the IPC structure across all levels of government and health facilities, which led to a standardized approach. This approach ensured that, in the face of COVID-19, healthcare workers felt protected, thus improving worker confidence and morale (*13*). The multimodal approach was shown to improve hand hygiene and other IPC practices in a cross-sectional survey of 17 hospitals in Greece (*14*). Similar sustainable improvements in hand hygiene were documented by Allegranzi et al. (*15*). Wang et al. (*16*) demonstrated that the COVID-19 pandemic highlighted the crucial role a structured IPC program plays in disease outbreak control. In 2020, WHO supported Ukraine to apply the multimodal approach in response to COVID-19 at the facility and national level, resulting in overall improvement of the IPC program (*17*).

Using standardized tools to assess health facilities enabled officials to identify gaps in IPC policy and guidelines implementation and create specific recommendations for remediation. Immediate targeted interventions were implemented at the facility level on the basis of the work plan. In addition, refresher trainings and national webinars were carried out on the basis of cross-cutting gaps. The WHO recommends use of IPC facility assessments to provide feedback and make IPC program improvements (*18*). It recommends implementing an assessment framework using a tool to assess 8 IPC core components that scores the IPC measures at the facility as inadequate, basic, intermediate, and advanced. Follow-up assessments should be conducted quarterly, semiannually, or annually. Although the baseline assessment mean score in Kenya of 61% was lower than the score of 86% documented in Germany, the difference could be accounted for by timing, setting, and tools used (*19*). Sachdeva et al. (*20*) showed varied compliance to IPC measures among 30 facilities in India. The assessment in Kenya demonstrated that the domains of hand hygiene and mask use scored the highest. This finding is likely because the 2 methods were being emphasized as the key COVID-19 prevention measures. Handling of human remains scored the lowest because no healthcare worker training had been held on that subject. Although risk for SARS-CoV-2 transmission through a dead body is minimal, standard precautions should be practiced and education offered to allay fears (*21*–*24*). Other domains that scored low included appropriate use of gloves, monitoring of healthcare workers, and care for COVID-19 patients, which might reflect a knowledge gap because of low access to training, lack of supportive guidance documents, and a shortage of gloves at the time.

In 2020, healthcare workers made up 3.2% of all SARS-CoV-2 infections in Kenya, which was lower than the global percentage of 3.9% (May 2020) and the percentages in Nigeria (6%), Italy (10%), and Spain (15%) (*25*–*29*) but higher than that reported in Singapore (1.7%) (*30*). The infection rate among HCWs in Kenya was 12 times higher than the general population and higher than the 5.5 times higher rate documented in Ontario, Canada (K.L. Shwartz et al., unpub. data, https://www.medrxiv.org/content/10.1101/2020.06.12.20129619v2), an indication that healthcare workers remained at higher risk for infection. Despite the high number of healthcare workers trained in early 2020, the infection rate remained high. Almost two thirds of the trainings were held for 1 day, which was inadequate to cover some practical topics, such as donning and doffing of PPE. Use of PPE in this period was inadequate or improper because of global shortages. Infrastructure renovations to address overcrowding of patients, ventilation, and hand hygiene facilities might have been slow to resolve. Healthcare workers were overstretched and had prolonged exposure to many patients (some of whom were asymptomatic) during the surge periods in June–August and October–December 2020 (Figure 2). Such reasons have been documented in China (*31*). A follow-up case control study was conducted in Kenya to explore reasons for the high infection rate. Preliminary findings indicated that lack of PPE and lack of IPC trainings were risk factors for infection (M. Njeru, unpub. data). In the absence of COVID-19 vaccines in Kenya at the time, other measures to protect healthcare workers were implemented, such as training, provision of appropriate PPE, and active screening and prompt quarantine or isolation of exposed or infected workers.

This review had several limitations. Simultaneously implementing many of the new interventions and obtaining accurate and timely reports from all facilities and counties was difficult during the pandemic. No active surveillance occurred among healthcare workers; only persons with laboratory-confirmed SARS-CoV-2 were included in the national list. Persons who were asymptomatic and not tested were not considered. In addition, although many guidance documents were developed at the national level, only about half had reached the assessed facilities. Time pressures were intense, and many mitigation activities were happening concurrently. Dissemination of all documents in development was not well streamlined. These factors would have delayed adoption of COVID-19 prevention measures at facilities. Despite these limitations, this article provides a broad picture of Kenya’s COVID-19 IPC response. While the measures were in response to COVID-19, they likely reduced transmission of influenza and other respiratory viruses, as was shown in the United States, Australia, Chile, and South Africa, as well as a reduction in diarrheal disease as demonstrated in Kenya (*32*,*33*).

Although some challenges occurred, the IPC multimodal approach was a practical response to the pandemic in Kenya. Consideration can be made for adoption of this approach based on a country’s context. Systems to monitor the effects of implementation and address emerging gaps should be put in place. This approach might reduce the effect of COVID-19 by protecting healthcare workers and patients in current and future pandemics.
